# Analysis of the Relationship Between Unilateral Posterior Crossbite and Alterations in the Eruptive Trajectory of Maxillary Canines, the Occlusal Plane, and the Inclination of the Labial Commissure

**DOI:** 10.3390/children12040437

**Published:** 2025-03-29

**Authors:** Eugenia Martin-Romanillos, Gonzalo Feijóo, Andrea Martín-Vacas, María Rosa Mourelle-Martínez, Nuria E. Gallardo-López, Antonia M. Caleya

**Affiliations:** 1 PhD Program in Dentistry Science, Faculty of Dentistry, Complutense University of Madrid, 28040 Madrid, Spain; eromamar@uax.es; 2 Facultad de Odontología, Universidad Alfonso X El Sabio, Villanueva de la Cañada, 28691 Madrid, Spain; amartvac@uax.es; 3 Department of Dental Clinical Specialties, Faculty of Dentistry, Complutense University of Madrid, 28040 Madrid, Spain; gfeijoo@ucm.es (G.F.); mrmourel@ucm.es (M.R.M.-M.); negallar@ucm.es (N.E.G.-L.)

**Keywords:** crossbite, canines, labial commissure, occlusal plane, orthodontics

## Abstract

Objectives: The aim was to establish whether there is a relationship between the presence of unilateral posterior crossbite (u-PCB) and the mesio-distal inclination of permanent upper canines, the angulation of the occlusal plane, or the labial commissure inclination in children. Methods: A cross-sectional association study was conducted. Panoramic radiography was used to measure the inclination of the canines and the occlusal plane, and frontal rest photography was used to measure the inclination of the labial commissure. The measurements were performed with tpsDig264 software version 2.25, 2016, Ecology & Evolution, SUNY), and the data obtained were analyzed with SPSS 22.0 software, using ANOVA and Kruskal–Wallis tests for the statistical analysis. Results: A total of 106 patients with u-PCB and 41 patients in the control group were studied. No statistically significant differences were observed in the inclination of the right and left canines in patients with u-PCB compared to patients in the control group. There is a relationship between the presence of u-PCB and the inclination of the occlusal plane. No statistically significant differences were observed in the inclination of the labial commissure in patients with u-PCB compared to patients in the control group. Conclusions: There is no relationship between the presence of u-PCB and the alteration in the eruptive trajectory of the permanent upper canines or in the inclination of the labial commissure. However, a relationship between the presence of u-PCB and altered occlusal plane inclination was found.

## 1. Introduction

The correct dental relationship is essential for the development of oral physiological functions, particularly mastication and ingestion [1]. Correct dental relationship must be established in all three spatial planes, known as vertical, sagittal, and transverse. At the transverse level, in permanent dentition, normal occlusion in the posterior sector occurs when the palatal cusps of the upper premolars and molars contact the triangular and central fossae of the lower premolars and molars. Therefore, when this relationship is altered, it is referred to as a transverse plane malocclusion, which may occur independently of the intermaxillary relationship in the vertical and sagittal planes [2].

Transverse malocclusion can be classified into posterior crossbite (PCB), edge-to-edge bite, and scissor bite, PCB being the most common [3,4]. PCB is the most common transverse malocclusion, and it is defined as a transverse malocclusion in which the buccal cusps of the upper premolars and molars occlude lingually to the buccal cusps of the lower premolars and molars [5]. Transverse malocclusions may affect either hemi-arches (bilateral), only one hemi-arch (unilateral), or a single tooth [1]. Most commonly, PCB develops unilaterally, and only one hemi-arch is affected [1], because typically it is secondary from a narrow maxilla. This may be due to genetic or environmental factors [most commonly oral habits] and, in most cases, a combination of both [4].

However, sometimes, occlusal interference is also present, a functional mandibular shift occurring toward a position that, although incorrect, is more comfortable for the patient, as it allows them to achieve maximum intercuspation [4,5,6,7]. PCB has a high prevalence in patients with primary dentition, Europe showing the highest prevalence (15.38%) [8,9], showing the same prevalence during primary and permanent dentition [9]. What initially begins as a mandibular shift eventually develops into a skeletal problem, leading to facial asymmetry due to functional, dentoalveolar, and skeletal adaptation to the deviation [10,11,12,13]. However, the mandible shows no morphological differences after expansion treatment and retention [14]. For this reason, early treatment of this type of malocclusion is essential [3,15].

The upper canine has anatomical characteristics that promote its anchorage in the alveolar bone and occupies a strategic location, making it a tooth of foremost importance in occlusion. It serves as a guide for lateral movement [16,17]. However, it is relatively common for the upper canine to present alterations in its eruption path due to various causes, such as maxillary compression. Consequently, it could also be associated with crossbite. Excluding the third molars, the upper canine is the tooth that most often exhibits anomalies in its eruption [18,19]. At around the age of 9 or 10 years, swelling should appear in the vestibular area at the level of the primary canine, where the permanent canine can be palpated, showing that it will erupt later [20]. In the absence of this swelling, a sign of the canine eruption anomaly should appear [18,21,22]. Another sign that should raise concern is the persistence of the primary canine in the mouth [without mobility] in patients aged 14 or 15 years [23]. The position of the permanent upper canine can be determined by taking measurements using orthopantomography. Additionally, an occlusal radiograph is also captured to determine its vestibular or palatal position. However, nowadays, to accurately locate its position, CBCT (Cone Beam Computed Tomography) is preferable, due to its 3D characteristics. Nonetheless, we must not forget its drawbacks, such as its high cost and significant radiation exposure to the patient during the procedure [23,24,25,26].

The relationship between the alteration in the eruption of the permanent upper canines and the presence of narrow arches, compressed maxillae, and negative skeletal-to-dental discrepancies has been confirmed in multiple studies, such as those by Barros et al. and Montes-Díaz et al., among others [20,23]. In addition, according to Schindel [17], in 85% of impacted canine cases, the maxilla has normal dimensions. This research could aid in diagnosis and improve the prediction of potential retention or impaction of the permanent upper canine. On the other hand, when studying an individual’s facial aesthetics, symmetry is one of the most relevant traits, as it involves the balance between the shape, size, and position of their craniofacial structures. There are several factors that can alter facial symmetry, such as genetic, environmental, functional, and developmental factors. Crossbite accompanied by mandibular deviation often leads to facial asymmetry and alterations in the labial commissure, which can be seen and directly measured on the individual’s face [27].

The main aim is to establish whether there is a relationship between the presence of unilateral PCB and the mesio-distal inclination of permanent upper canines, the angulation of the occlusal plane or the labial commissure inclination in children.

## 2. Materials and Methods

A descriptive, observational, and cross-sectional study was designed, with a cross-variable association approach. Prior to the initiation of this study, approval was obtained from the Bioethics Committee of Clinic San Carlos Hospital (code 24/463-E). All applicable national and European regulations about the protection of personal data were strictly adhered to during the research.

### 2.1. Sample Description

The study population consisted of children aged 6 to 10 years who attended the Oral and Maxillofacial Diagnostic Centre in Madrid, Spain, seeking an occlusion assessment for orthodontic diagnostic purposes. The evaluation included a panoramic radiograph, lateral cephalometric radiograph, and a series of extraoral and intraoral photographs. Children with unilateral posterior crossbite (u-PCB), both right (r-PCB) and left (l-PCB), were included in this study. Exclusion criteria included congenital anomalies, systemic diseases, head and neck deformities, prior or current orthodontic/orthopedic treatment, other types of malocclusions, cavitated caries, dental morphology or structural abnormalities, and dental agenesis. This study was conducted between September and December 2024, using the centre data base to filter patient reco1rds of children aged between 6 and 10 years, independent of data records. Secondarily, the IP excluded subjects with other malocclusions apart from u-PCB, according to inclusion–exclusion criteria.

The sample size was calculated using G*Power software (version 3.1.9.7) with the ANOVA procedure for repeated measures, assessing intra- and intergroup interactions [28,29]. With an effect size of 0.41 (according to SPSS), an alpha error of 0.05, and a power of 80%, a total of 63 subjects would be required to perform comparisons involving three groups and two related measurements. To account for potential dropouts, the required sample size was increased by 20%, resulting in a planned sample size of 76 subjects (38 subjects per subgroup). A non-probabilistic sampling method was used, including all children who met the predefined eligibility criteria.

The patients’ ages were recorded, classifying them into four categories (6 years, 7 years, 8 years, and 9 years). The sex of the subjects was also recorded, in order to analyze differences associated to sexual dimorphism.

### 2.2. Study Procedure

The patients were grouped into three categories based on the dependent variable, u-PCB. Group 1 was established as the control group (no posterior crossbite), and groups 2 and 3 were composed of children with r-PCB and l-PCB, respectively. The evaluated variables included the inclination of the permanent maxillary canines (MaxC-inc) and the occlusal plane (OccPl-inc) compared to the midline of the lower facial third, as well as the inclination of the labial or intercommissural line (IntComm-inc) relative to the facial midline.

The radiographic measurements of canine inclination and the occlusal plane were performed using orthopantomographies. All radiographs were taken with the same X-ray device (Siemens Orthopantomograph, Ortofox^®^) and by the same operator. Measurements were conducted by a single operator using tpsDig2 software (tpsDig264, version 2.25, 2016, Ecology & Evolution, SUNY). The radiographs were analyzed on a 13” monitor with the zoom set to 100%, and in cases of uncertainty, the image was enlarged to 110%. A maximum of 30 radiographs were analyzed per session, and all radiographs were obtained under the same technical specifications (nominal voltage = 208/220/230/240 V, rated current = 12 A, frequency = 50/60 Hz, tube current = 9–16 mA, aluminum equivalent filter = 2.5, focal spot size = 0.5 × 0.5 mm, standard technique used = 65 kV/12 mA, fixed exposure time = 12 s).

**Figure 1:**
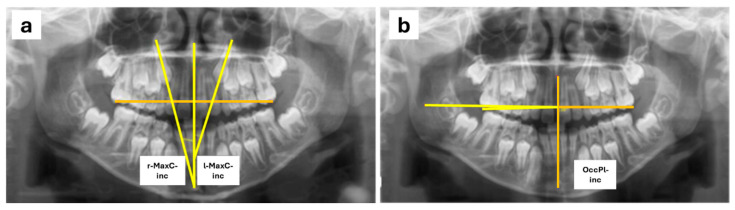
Radiographic measurements conducted in orthopantomographies: MaxC-inc (**a**) and OcclPl-inc (**b**).

For the measurements, a reference plane was set up using the midline passing through the midpoint of the palatal suture and the anterior nasal spine. To assess MaxC-inc, the angle between the reference plane and a line following the longitudinal axis of the maxillary canines was measured (Figure 1a) following the Power–Short and Canut techniques [30,31]. For the measurement of OccPl-inc, the occlusal plane was defined by drawing a line passing through a point at the center of the occlusal surface of the permanent first maxillary molars or, if the permanent molars had not yet erupted, the second maxillary primary molars. A line perpendicular to the reference plane was drawn at the point where it intersected the occlusal plane on the right side of the patient, and the angle formed between this perpendicular to the midline and the occlusal plane was measured (Figure 1b). Also, right (r-) and left (l-) sides were evaluated.

For the measurement of IntComm-inc, a frontal resting photograph was used. The reference plane was defined as the facial midline, drawn by connecting the glabella and subnasal points and extending it to the chin. The intercommissural line was traced by connecting both labial commissures. A line perpendicular to the facial reference plane was then drawn, and the angle formed between this perpendicular and the intercommissural line was measured.

### 2.3. Blinding

The data were collected anonymously from the database of the aforementioned radiographic diagnostic center. Therefore, the measurements and data analysis were conducted in a blinded manner by both the operators and the data analyst. All measurements were performed by the same operator (E.M.-R.). To evaluate the accuracy of the method, a second measurement was taken from a 10% random sample of the studied subjects, performed by the same operator and by a second operator (A.M.C.).

### 2.4. Statistical Analysis

Data analysis was performed using SPSS software for Windows (version 22.0, Armonk, NY, USA), with a 95% confidence level (*p* ≤ 0.05) and asymptotic or bilateral significance. Intra- and inter-operator concordance was evaluated in a 10% random sample of the study subjects using the intraclass correlation coefficient (ICC), interpreted according to the Landis and Koch scale [32]. For sample description, the frequency and percentage of qualitative measures (sex and crossbite) were calculated, and the mean and standard deviation of quantitative measures (age, MaxC-inc, OccPl-inc, IntComm-inc) were determined. A Student’s *t*-test was performed to analyze differences between crossbite sides (right and left) or between sexes. A parametric ANOVA test was conducted to evaluate the hypothesis.

## 3. Results

Of the 355 subjects initially identified, a final sample of 106 patients with u-PCB and 41 control subjects was obtained (Table 1). The normality of the studied variables was assessed, and it was found that the variables met the normality criteria in the overall sample, although this was not always the case for the distribution by age, sex, or crossbite. As a result, parametric tests were selected for hypothesis testing.

Prior to the start of this study, intra- and inter-operator concordance was analyzed (Table 2) to validate the measurements performed, resulting in almost-perfect to perfect agreement for the analyzed variables, except for the intra-observer OccPl-inc (acceptable), the inter-observer r-MaxC-inc (acceptable), and the inter-observer OccPl-inc (moderate).

### 3.1. Relationship Between the Inclination of the Canines and the Presence of Unilateral Posterior Crossbite

According to our results, the differences in Max-C-inc between children with u-PCB and the control group were not statistically significant (Table 3) (*T*-test *p* > 0.05), although there was a tendency to observe a greater inclination in the canine on the side of the crossbite. A variable was also created to measure the difference in MaxC-inc between the right and left canines, and whether there were differences in canine inclination among subjects with r-PCB (difference = 0.18 ± 7.47, *p* = 0.854) or l-PCB (difference = 0.54 ± 5.42, *p* = 0.490). However, significant differences were found in the control group (difference = 2.36 ± 5.62, *p* value = 0.01), with greater inclination in the left canine (11.40°) compared to the right canine (9.04°). This difference in contralateral canine inclination suggests that, although no statistical differences were found between the PCB and control groups, children without transverse malocclusion showed an asymmetry in Max-C-inc; however, no differences were obtained in the u-PCB group or between study groups.

The influence of age, sex, and u-PCB on the inclination of the canine homologous to the PCB or contralateral canine was analyzed (Table 4). The results indicate that, although age does affect the MaxC-inc in both cases (ANOVA *p* = 0.001 and *p* = 0.004), the combined evaluation of age, sex, and u-PCB does not significantly alter the inclination of the canines.

A more detailed analysis was conducted on the influence of age on canine inclination, without considering sex (Table 5). Significant differences in MaxC-inc were found between the 6- and 8-year-old groups in both the control group and in the u-PCB groups in homolateral and contralateral canines (Bonferroni *p* = 0.005, *p* = 0.005, and *p* = 0.044, respectively). There was a significant increase in MaxC-inc in the 8-year-old group compared to the 6-year-old group. The mean differences (−4.32° ± 1.27, −7.33° ± 2.10, and −6.42° ± 2.34, respectively) indicate a significant increase in angulation at 8 years of age compared to the younger group, which then corrects by the age of 9 years. No significant differences were found between the other age categories.

The influence of gender was studied, showing a similar mean inclination in both females and males, with no significant differences (ANOVA *p* > 0.05).

### 3.2. Relationship Between the Inclination of the Occlusal Plane and the Presence of Unilateral Posterior Crossbite

The relationship between u-PCB and the OccPl-inc was analyzed, showing that the OccPl-inc was significantly lower in children with u-PCB (−0.25° ± 1.74) compared to the control group (0.44° ± 1.85) (*T*-test *p* = 0.037). The influence of age, sex, and u-PCB on the OccPl-inc was studied, with no statistically significant differences observed (ANOVA *p* > 0.05) (Table 6).

### 3.3. Relationship Between the Inclination of the Labial Commissure and Unilateral Posterior Crossbite

The IntComm-inc was analyzed in relation to u-PCB, and it was found that the angulation was similar between the crossbite group (−1.23° ± 3.28) and the control group (−0.55° ± 2.06) (*T*-test *p* = 0.220). No significant differences were found when differentiating the location of the crossbite (right side: −1.32° ± 3.99 vs. left side: −1.12° ± 2.24) compared to the control group nor between the two sides of the u-PCB (ANOVA *p* > 0.05).

The influence of age on the IntComm-inc was analyzed, and no significant differences were found between the age categories studied (ANOVA *p* > 0.05). A trend was observed showing a progressive increase in inclination between the ages of 6 and 8, followed by a decrease at 9 years, although no significant differences were noted (Table 7). Sex was also not found to be associated with the IntComm-inc, with no differences between boys (−0.94° ± 2.22) and girls (−1.10° ± 3.41) (ANOVA *p* > 0.05).

## 4. Discussion

The frequency of u-PCB is around 5% in the general population in Spain [3], with certain studies reporting it to reach up to 50% in orthodontic populations [3,33]. Similarly, alterations in the eruptive path of the permanent upper canines are common, being diagnosed in up to 3% of the general population, with these figures rising to 23.5% in patients seeking orthodontic treatment [21,33,34]. Facial symmetry is considered a balance between the shape, size, and position of the craniofacial structures. Several factors can contribute to altering this symmetry, such as PCB, which, in some cases, causes a deviation of the chin, leading to alterations in the labial commissure or the occlusal plane.

Our sample size was 147 patients; in other previous studies, sample sizes ranged from 21 to 1,282 subjects [21,35,36,37,38,39]. In relation to the radiographic measurements conducted, a modification of the Power–Short technique [30] and the Canut method [31] was carried out, similarly to previous authors [21,29,40,41]. Other authors used different reference planes to measure occlusal inclination, such as the infraorbitary [34] or Gonion–symphysis plane [26]. Intercommisural inclination was measured with respect to facial midline. Vicente and Mourelle studied facial asymmetry related to u-PCB, analyzing different facial areas [42]. Song et al. [39] used lineal measurements to analyze symmetry in non-malocclusion subjects.

Regarding the inclination of the canines, we can observe that our data significantly differ from those obtained by Sajnani et al. [21] in terms of the inclination angle of the non-impacted canine, particularly in the age groups of 6, 8, and 9 years, with differences reaching up to 8 degrees in the 6-year-old group. The discrepancy is smaller in the 7-year-old group. It is important to note that Sajnani did not classify patients based on their transverse occlusion; they did not differentiate between those with or without PCB, measuring only the inclination of the canines while distinguishing between impacted canines and those with normal eruption. In the case of the study conducted by Alqerban et al. [43], the average value of the angulations for impacted canines is 20.7°, while for non-impacted canines, it is 8.8°. When comparing with our results, our data more closely resemble those found by these authors for non-impacted canines. Similarly, when comparing our results with those obtained by Ticona and Diéguez [36], we observe that these authors found a greater inclination of the canine on the PCB side (13.88° compared to 11.75° on the non-crossbite side), while in our case, the average inclination on the crossbite side was 10.51°, with a mean of 10.61° on the non-crossbite side. These differences were not statistically significant.

In relation to the inclination of the occlusal plane, we found a similar study conducted by Uesuge et al. [40], in which they analyze the inclination of the occlusal plane in adult patients with mandibular deviation. These authors found a tendency for the occlusal plane to incline toward the side of the mandibular deviation, except in 25% of the patients, where the inclination of the occlusal plane occurred toward the contralateral side of the mandibular deviation. In our study, we found comparable results, indicating a significant relationship between the presence of PCB and the inclination of the occlusal plane.

Similarly, Padwa et al. [41] studied the relationship between facial asymmetry and the presence of occlusal plane canting. They divided the sample of patients based on whether they had facial asymmetry or not, using extraoral photographs. These authors determined that, in the analysis performed by experienced observers, 90% of patients with facial asymmetry had occlusal plane canting, whereas only 27% of patients in the non-asymmetry group showed the same feature. In contrast, observers without experience found 82% and 30% of patients with occlusal plane canting in the asymmetry and non-asymmetry groups, respectively. Similarly to our case, they found a relationship between the presence of facial asymmetry and occlusal plane canting. In our study, we also found statistically significant differences, although we analyzed the inclination of the occlusal plane in pediatric patients with u-PCB, who lacked evident skeletal facial asymmetry, although they presented some degree of functional asymmetry due to the presence of u-PCB.

Regarding the inclination of the labial commissure, we found a study analyzing the relationship between the presence of u-PCB and alterations in facial symmetry, conducted by Vicente et al. [42] in Madrid, which yielded the same results as ours. There were no statistically significant differences between the PCB side and the non-crossbite side. However, while we analyzed the angulation of the labial commissure, they studied facial areas without focusing on any specific lines. Similarly, they compared sexes by dividing the participants based on whether they had u-PCB or not, finding no statistically significant differences between the groups of children, which is consistent with our results.

According to the results of our study, and comparing them with the findings from similar studies, it can be stated that, although unilateral posterior crossbite exists and it can affect the inclination of the occlusal plane, there seems to be compensation by the soft tissues, as the inclination of the labial commissure does not appear to be affected.

This study presents some limitations, taken into account in the data interpretation. First of all, orthopantomography has a degree distortion and magnification that varies depending on the X-ray device [44,45], greater in the horizontal plane [46] due to it being a two-dimensional evaluation. However, the use of these radiographs has been shown to be also valid for performance of measurements [46,47,48], other factors associated to the patient (malocclusions, bone asymmetries, etc.), or radiographic technique. On the other hand, radiograph distortion has been observed to be greater in the anterior region than in the posterior one [48].

This limitation was avoided by always using the same X-ray machine and the same operator. This decision also minimizes possible biases in terms of patient positioning or image quality, considering that all the images were taken under the same conditions and the same environment, carried out by only one operator. The homogeneous distribution of the sample in terms of race and age makes our results reliable.

Interceptive orthodontics is of great relevance, because early treatment of dental malocclusions prevent severe malocclusions in permanent dentition. Also, due to the results, correcting dental occlusion prevents aesthetic alterations. It would be interesting to conduct similar research in children under 6 years and the special care population. Moreover, a recent systematic review has stated the negative influence of malocclusion on oral ingestion, suggesting that orthodontic treatment plans should aim to enhance oral function [49].

## 5. Conclusions

Based on our results, we can conclude that there is no relationship between the presence of unilateral posterior crossbite and alteration in the eruptive trajectory of the permanent upper canines or in the inclination of the labial commissure, which indicates that there is soft tissue compensation in patients with unilateral posterior crossbite, although further studies are needed to confirm this. However, there does appear to be a relationship between the presence of unilateral posterior crossbite and altered occlusal plane inclination.

## Figures and Tables

**Table 1:** Sample distribution according to demographic outcomes.

Age	Total		Male		Female	
	N	%	N	%	N	%
6	61	41.5	18	12.2	43	29.3
7	38	25.8	17	11.6	21	14.2
8	26	17.7	11	7.5	15	10.2
9	22	15	11	7.5	11	7.5
TOTAL	147	100	57	38.8	90	61.2

**Table 2:** Intra- and inter-observer ICC evaluation.

	Intra-Observer		Inter-Observer	
	ICC	Sig	ICC	Sig
r-MaxC-inc	0.874	<0.001 *	0.571	0.026 *
l-MaxC-inc	0.937	<0.001 *	0.642	0.012 *
OccPl-inc	0.283	0.186	0.442	0.178
IntComm-inc	0.630	0.013 *	0.913	<0.001 *

* Sig: statistically significant, *p* < 0.05.

**Table 3:** Description of maxillary canines’ inclination in study and control samples, with significance values of *T*-test. LL, lower level. UL, upper level.

	r-PCB			Control			Sig
	Mean ± SD	CI 95%		Mean ± SD	CI 95%		
		LL	UL		LL	UL	
**r-MaxC-inc**	**10.22 ± 7.93**	**8.16**	12.28	9.04 ± 8.15	6.55	11.53	0.475
**l-MaxC-inc**	9.88 ± 8.04	7.79	11.31	11.41 ± 7.20	9.21	13.61	0.334
	**l-PCB**			**Control**			**Sig**
	**Mean ± SD**	**CI 95%**		**Mean ± SD**	**CI 95%**		
		**LL**	**UL**		**LL**	**UL**	
**r-MaxC-inc**	10.81 ± 7.95	8.58	13.04	9.04 ± 8.15	6.55	11.53	0.30
**l-MaxC-inc**	11.35 ± 7.78	9.17	13.53	11.41 ± 7.20	9.21	13.61	0.971

**Table 4:** Summary of ANOVA significance levels of studied outcomes evaluating MaxC-inc.

Summary	Canine Homolateral to the u-PCB	Canine Contralateral to the u-PCB
	Sig	Sig
**Age**	0.001 *	0.004 *
**Age-Sex**	0.370	0.23
**Age-PCB**	0.140	0.30
**Sex-PCB**	0.540	0.32
**Age-Sex-PCB**	0.510	0.10

* Sig: statistically significant.

**Table 5:** Canine inclination in the control group and u-PCB groups: homolateral and contralateral canine relative to the u-PCB. LL, lower level. UL, upper level.

Age	Control			MaxC-inc Homolateral to the u-PCB			MaxC-inc Contralateral to the u-PCB		
	Mean ± SD	CI 95%		CI 95%	CI 95%		Mean ± SD	CI 95%	
		LL	UL		LL	UL		LL	UL
**6**	**8.71 ± 7.62**	**7.34**	10.07	8.10 ± 7.11	6.08	10.12	8.01 ± 8.03	5.73	10.30
**7**	10.67 ± 7.48	8.96	12.38	11.21 ± 7.30	8.38	14.04	13.42 ± 9.71	9.66	17.19
**8**	13.04 ± 7.21	11.03	15.04	15.44 ± 5.17	12.68	18.19	14.44 ± 7.78	10.29	18.58
**9**	11.79 ± 8.79	9.11	14.46	14.38 ± 10.79	7.52	21.24	9.57 ± 7.14	5.03	14.10

**Table 6:** Summary of ANOVA significance levels of studied outcomes evaluating OccPl-inc.

Summary	Sig
Age-Sex	0.323
Age-PCB	0.409
Sex-PCB	0.288
Age-Sex-PCB	0.996

**Table 7:** Description of intercommisural inclination in the age categories. LL, lower level. UL, upper level.

Age	IntComm-inc		
	Mean ± SD	CI 95%	
		LL	UL
6	−1.42 ± 3.70	−2.37	−0.74
7	−0.87 ± 2.51	−1.69	−0.04
8	0.01 ± 2.04	−0.81	0.84
9	−1.51 ± 2.31	−2.54	−0.49

## Data Availability

The raw data presented in this study are only available on request from the corresponding author due to privacy restrictions.

## Abbreviations

The following abbreviations are used in this manuscript:

PCB	Posterior crossbite
MaxC-inc	Inclination of the permanent maxillary canines
OccPl-inc	Inclination of the occlusal plane
IntComm-inc	Inclination of the labial or intercommissural line
